# Insider knowledge as a double-edged sword: an integrative review of midwives’ personal childbearing experiences

**DOI:** 10.1186/s12884-022-04962-y

**Published:** 2022-08-15

**Authors:** S. Coulton Stoliar, H.G. Dahlen, A. Sheehan

**Affiliations:** School of Nursing and Midwifery, Parramatta South Campus, Western Sydney University, NSW, Parramatta, Australia

**Keywords:** Childbirth, Pregnancy, Midwives, Childbirth experience, Midwife mothers

## Abstract

**Background:**

The majority of maternity care is provided by female midwives who have either become mothers or are of childbearing age, but there is limited research exploring midwives’ own personal childbearing experiences. This integrative review aims to explore the published literature and research on midwives’ own experiences of pregnancy and childbirth.

**Method:**

An integrative review of the literature was conducted after relevant articles were identified through a search of: five electronic databases (Cumulative Index of Nursing and Allied Health Literature (CINAHL), Medline, PubMed, Scopus, and Google Scholar), cited reference lists, and networking with peers. Similar and contrasting patterns and relations within the literature were identified and grouped into themes and subthemes.

**Results:**

Twenty articles were included in the review and four overarching themes were identified. *Insider knowledge plays a role in decision making* encompassed the way midwives used their knowledge to choose; a preferred mode of birth, maternity care provider, model of care, and place of birth. *Navigating the childbirth journey* demonstrated how some midwives were able to use their insider knowledge to achieve agency, while others had difficulty achieving agency. This theme also revealed the ‘midwife brain’ that midwives need to manage during their childbearing journey. The theme *impact of care on the birth experience* described how the type of care the midwives received from maternity care providers affected their overall birth experience. The fourth theme *from midwife to mother* explains their preparedness for childbirth and their transition to motherhood.

**Conclusion:**

For childbearing midwives, there is a potential conflict between their position as knowledgeable experts in maternity care, and their experience as mothers. Whilst they can use their insider knowledge to their advantage, they also experience heightened fear and anxiety through their pregnancy. It is important for maternity care providers to acknowledge and support them and provide balanced and tailored care that acknowledges the woman within the professional midwife and the professional midwife within the woman.

## Background

Childbirth has been described as a multidimensional and profound experience that can have both short and long-term physical, psychological, social, and existential impacts on women [1–11]. Research has identified that the way women are cared for during pregnancy and the birth of their child, can impact on their overall childbearing experience. Women who report being well supported during their childbirth journey have described childbirth positively, as a moment of triumph, satisfaction, and reward [9, 12–14]. In contrast to this, women have also reported negative experiences of childbirth, with some women describing their birthing experiences as dissatisfying or even traumatic [6–8, 15, 16]. Most of the literature surrounding pregnancy and birth experiences focuses on women’s experiences of birth [17], or midwives’ professional experiences of caring for women during births [18].

The majority of maternity care is provided by female midwives who have either become mothers or are of childbearing age [19, 20], yet there is limited research exploring midwives’ own personal childbearing experiences. Previous research has found that personal birth experiences can influence the professional practice of maternity care providers, however this research was conducted on obstetric nurses in the United States of America (USA) [21]. The purpose of this integrative review was to explore the published literature and research on midwives’ own experiences of pregnancy and childbirth. This review did not explore the influence of midwives’ personal child birthing experience on their professional practice.

## Methods

An integrative review allows for the inclusion of various sources of literature on a topic including research from various methodological paradigms [22–24]. An integrative review is considered able to provide a more holistic understanding of a phenomenon of interest than other review methods [24]. As the aim of this review was to gain a broad understanding of the childbirth experiences of midwives, an integrative review using Wittemore and Knafl’s [24] five stage framework (problem identification, literature search, data evaluation, data analysis, presentation) was undertaken.

### Search strategy

A comprehensive keyword search of the literature was conducted on five databases between 1980 and 2021: Cumulative Index of Nursing and Allied Health Literature (CINAHL), Medline, PubMed, Scopus, and Google Scholar. Search terms and variations of search terms using Medical Subject Headings (MeSH terms) included, but were not limited to: ‘pregnancy,’ ‘childbirth,’ ‘parturition,’ ‘birth experience’, ‘midwives’, ‘midwifery,’ ‘personal experience’, ‘life experience,’ ‘life change events,’ and/or ‘personal narratives.’ A search of cited reference lists, and networking with peers were additional strategies used to search the literature. Due to the paucity of research on this topic, no time limit was place on publication of literature. All research articles were limited to primary studies with participants who were midwives or nurse-midwives. There was one primary study that included both midwife and nurse participants [25] that was also included. The findings in this particular study did not always differentiate between the midwife and nurse participants, however when it did, data clearly relevant to midwife participants was included in this integrative review. No review studies on this topic were identified. The inclusion and exclusion criteria are detailed in Table 1.

**Table 1:** Inclusion and exclusion criteria

**Inclusion criteria**	**Exclusion criteria**
• Primary qualitative, quantitative studies about the personal pregnancy and birth experiences of midwives • Studies must include participants who are midwives or nurse-midwives in their sample • Articles published in English • Research and anecdotal records must be published in peer-reviewed or non-peer reviewed professional journals	**•** Studies that reported the pregnancy and birth experiences of student midwives, birth assistants, doulas, or obstetric nurses **•** Published in a language other than English • Anecdotal and research papers reporting birth experiences of women before they completed their midwifery training **•** Conference abstracts and letters to the editor that did not publish results **•** Birth stories published on website blogs

### Search results

**Fig. 1:**
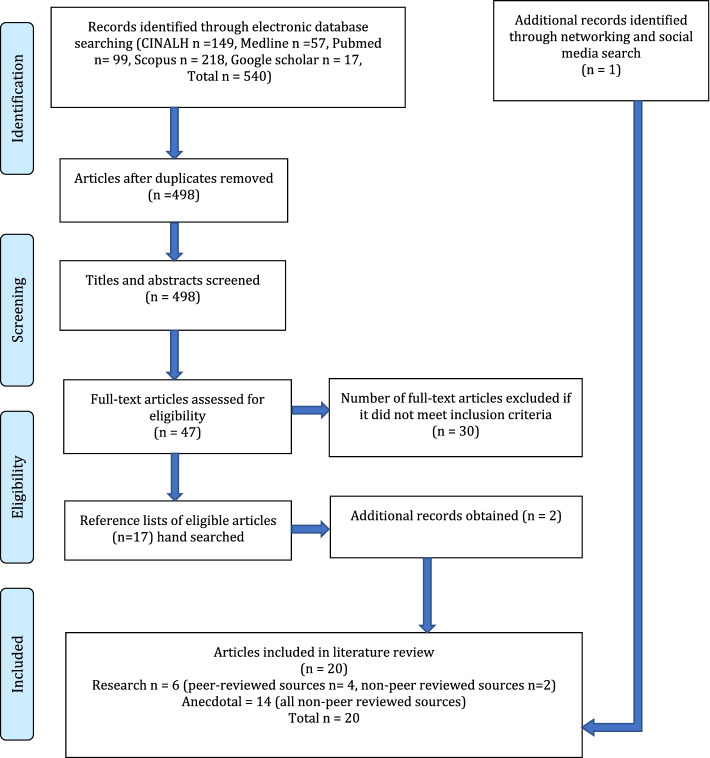
Systematic search results

A total of 20 articles, (six research and 14 anecdotal articles), were included in this integrative review after eliminating duplicates, and applying the inclusion /exclusion criteria (Table 1). The literature search strategy used for the review is presented in the Preferred Reporting Items for Systematic reviews and Meta-Analysis (PRISMA) flow diagram [26] (see Fig. 1).

### Evaluation of the literature

Quality appraisal of the literature was undertaken using the Critical Appraisal Skills Programme (CASP) tool [27]. Table 2 displays the methodological quality of the qualitative studies and Table 3 displays the methodological quality of the quantitative studies. As the review sought to explore all evidence relating to the research question, and due to a paucity of published research on this topic, no disqualifications were made on the grounds of quality as it was determined that valuable information may be lost if studies were excluded.

**Table 2:** CASP methodological quality appraisal of qualitative studies

**CASP Item**	**CASP Key**	**Church (2014)** [28]	**Redwood (2008)** [25]
**1**	Was there a clear statement of the aims of the research?	Yes	Yes
**2**	Is a qualitative methodology appropriate?	Yes	Yes
**3**	Was the research design appropriate to address the aims of the research?	Yes	Yes
**4**	Was the recruitment strategy appropriate to the aims of the research?	Unclear^a^	Yes
**5**	Was the data collected in a way that addressed the research issue?	Unclear^b^	Unclear^b^
**6**	Has the relationship between the researcher and the participants been adequately considered?	Yes	Unclear
**7**	Have ethical issues been taken into consideration?	Yes	Unclear^c^
**8**	Was the data analysis sufficiently rigorous?	Yes	Yes
**9**	Is there a clear statement of findings?	Unclear^d^	Yes
**10**	How valuable is the research?	Clear	Clear

^a^Did not explicitly state how participants were selected

^b^Theoretical saturation not discussed

^c^Have not discussed how they handled the effects of the study on participants

^d^Credibility of findings not explicitly discussed

**Table 3:** CASP methodological quality appraisal of quantitative studies

**CASP Item**	**CASP Key**	**Battersby (2002)** [29]	**Leinweber et al. (2017)** [30]	**McMulkin & Malone (1994)** [31]	**Toohil et al. (2019)** [32]
**1**	Did the study address a clearly focused issue?	Yes	Yes	Yes	Yes
**2**	Were the participants recruited in an acceptable way?	Yes	Yes	Yes^a^	Yes
**3**	Was the exposure accurately measured to minimise bias?	N/A	N/A	N/A	N/A
**4**	Was the outcome accurately measured to minimise bias?	Unclear^	Yes	Unclear^b^	Clear
**5a**	Have the authors identified all important confounding factors?	N/A	N/A	N/A	N/A
**5b**	Have they taken account of the confounding factors in the design and/or analysis?	N/A	N/A	N/A	N/A
**6**	A) Was the follow up of subjects complete enough? B) Was the follow up of subjects long enough?	N/A	N/A	N/A	N/A
**7**	What are the results of this study?	Clear	Clear	Clear	Clear
**8**	How precise are the results?	Unclear	Clear	Unclear	Clear
**9**	Do you believe the results?	Yes	Yes	Unclear	Yes
**10**	Can the results be applied to the local population?	Yes	Yes	Yes	Yes
**11**	Do the results of the study fit with other available evidence?	Yes	Yes	Yes	Yes
**12**	What are the implications of this study for practice?	Clear	Clear	Clear	Clear

^a ^Convenience sample

^b^ Reliability and validity of the questionnaire not reported

### Data extraction, reduction and analysis

In order to extract and collate all relevant information, tables were initially used to list details and characteristics of each article (see Tables 4 and 5). For a more focused and detailed reduction of each of the literature sources [22] the text from each article was loaded into Quirkos to conduct the qualitative analysis of the literature. Using the process of constant comparison, initial codes from the literature relating to midwives’ personal pregnancy and childbearing experiences were identified and were compared and contrasted to observe for patterns and themes. Continued use of constant comparison identified similar and contrasting patterns and relationships, which were ultimately grouped into themes and subthemes. Themes and subthemes were discussed and refined until consensus was achieved between all authors.

**Table 4:** Descriptive characteristics of the included studies

**Author**	**Year** **(Location)**	**Title**	**Record type**	**Study design**	**Sample**	**Data collection**
**Battersby** [29]	2002 (UK)	Midwives' embodied knowledge of breastfeeding	Non peer-reviewed research	Descriptive explorative quantitative study	410 midwives (results are based on the 307 midwives who reported personal experience of breastfeeding)	Surveys
**Church** [28]	2014 (UK)	Midwives' personal experiences of pregnancy and childbirth: Exploring issues of autonomy and agency in relation to the use of professional knowledge	Peer-reviewed research	Qualitative reflexive auto/biographical study	Nine midwives who had completed their training prior to becoming mothers	Interviews
**Leinweber, et al** [30]	2017 (AUS)	A socioecological model of posttraumatic stress among Australian midwives	Peer-reviewed research	Descriptive cross-sectional study	601 midwives	Survey
**McMulkin & Malone** [31]	1994 (UK)	Breastfeeding—midwives' personal experiences	Non peer-reviewed research	Descriptive cross-sectional quantitative study	210 midwives who had personal experience of breastfeeding	Questionnaire (structured and semi-structured questions)
**Toohill et al** [32]	2019 (AUS)	Trauma and fear in Australian midwives	Peer-reviewed research	Descriptive cross-sectional quantitative study	249 midwives	Online survey containing fixed response and free text questions
**Redwood** [25]	2008 (UK)	Exploring changes in practice: when midwives and nurses become mothers	Peer-reviewed research	Qualitative phenomenological study	Phase one: 22 midwives and nurses in their second and third trimesters of pregnancy. Phase two: interviews conducted at 12 months following childbirth (20 participants had returned to work by then)	Interviews

**Table 5:** Descriptive characteristics of the anecdotal literature

**Author** **Year/Location**	**Title**	**Number of births**	**Mode of birth**	**Place of birth**	**Maternity care provider**	**Experience with maternity provider(s)**	**Overall birth experience**
**Berkley** **2002 (UK)** [33]	A midwife's reflection on a homebirth: thoughts of a midwife becoming a mother	1	NVB	Home	Homebirth midwives	Positive	Positive
**Burlow** **1999 (UK – Scotland)** [34]	A midwife to myself	1	NVB	Home	Community midwives (Hospital midwives)^a^	Negative	Traumatic
**Constable** **2011 (UK)** [35]	Switching off the ‘Midwife’	1	NVB	Home	Homebirth midwife (also a friend)	Positive	Not stated
**Cooke** **2010 (location unknown)** [36]	A better midwife?	1	NVB	Hospital	Hospital midwives	Not stated	Positive
**Duggan** **1997 (UK -England)** [37]	Professional: How will it change your practice?	1	NVB	Home	Midwife friends	Positive	Positive
**Hinsliff** **2010 (location unknown)** [38]	Torn in two: birth decisions after a third degree tear	2	NVB (unplanned). Planned elective caesarean birth	Not stated	Not stated	Not stated	Positive
			Elective caesarean	Hospital	Hospital midwives and private obstetrician	Positive	Positive
**Jennings** **2005 (UK)** [39]	Midwife as mother, midwife as client	1	NVB	Home	Homebirth midwives	Positive	Positive
**Knapp** **2013 (CAN)** [40]	Rebirth	2^b^	NVB (VBAC)	Home	Homebirth midwives	Positive	Positive
**Lee-Ribas** **2008 (USA)** [41]	Face to Face: A Midwife’s Birth Story	1	NVB	Home	Homebirth midwives	Negative	Traumatic
**Moes** **2004 (USA)** [42]	Giving birth: A Midwife’s Faith in Birth is Reborn	1	NVB	Home	Homebirth midwives (also her friends)	Positive	Positive
**Neiger** **2004 (UK)** [43]	Choices and Changes	1	NVB	Home	Independent homebirth midwives	Positive	Positive
**South** **2016 (UK)** [44]	Birthing Instincts or a Midwife’s Intuition?	1	NVB	Home	Homebirth midwives (also her friends)	Positive	Positive
**Tennant** **1982 (location unknown)** [45]	Never the Same Again	1	Not stated	Hospital	Hospital midwives	Not stated	Positive
**Wilde** **2004 (location unknown)** [46]	Only Connect	2	NVB	Home	Homebirth midwives	Positive	Positive
			NVB	Hospital	Hospital	Negative	Neutral

*NVB* Normal vaginal birth

*VBAC* Vaginal Birth After Caesarean

^a^birth was assisted by a midwife friend not registered to practice in UK

^b^only the second birth experience which occurred after she obtained her midwifery qualification has been included in this review

## Results

Twenty papers were included in this integrative review, six research articles and 14 anecdotal articles. The six research articles were studies conducted in either Australia or the United Kingdom. Two studies used a qualitative approach [25, 28], and four a quantitative methodology [29–32]. All 14 anecdotal papers were published in professional midwifery journals.

**Fig. 2:**
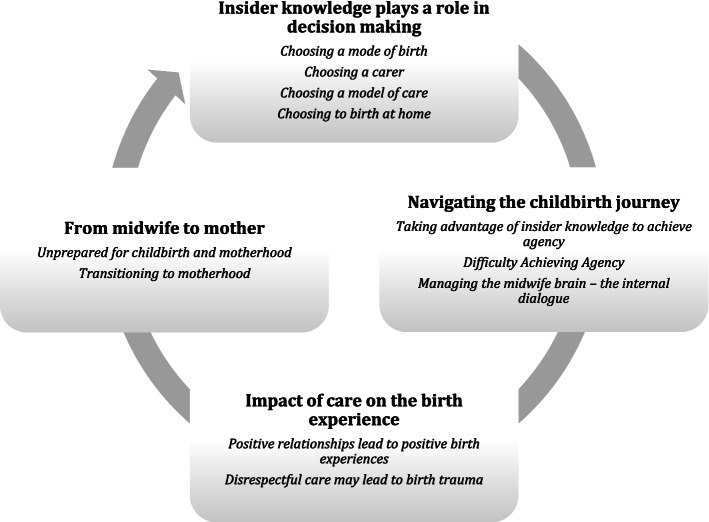
Mapping of themes and subthemes

Four overarching themes were identified: ‘Insider knowledge plays a role in decision making’, ‘Navigating the childbirth journey’, ‘Impact of care on the birth experience’, “From midwife to mother’ (see Fig. 2).

### Theme 1: Insider knowledge plays a role in decision making

This theme included four subthemes: ‘choosing a mode of birth’; ‘choosing a carer’; ‘choosing a model of care’; and ‘choosing to birth at home’.

It was apparent from this review that midwives were aware that they possess unique knowledge about obstetric risks, and various medical conditions and complications that could occur, when compared to women in general [28, 29, 31, 33, 35, 38, 44]. This ‘insider knowledge’ played a key role in their decision making and was used to actively make decisions that would allow them to be in control of their care [28, 37, 41, 43, 44, 46]. Midwives made choices to achieve control over external factors, such as the birthing environment, who their maternity care provider was, and the type of interventions they would allow [33–35, 37, 39, 41, 42, 44]. Being in control was particularly important for midwives who had non-conventional birth plans [40, 41, 43, 44].

#### Choosing a mode of birth

For the majority of midwives in this review there was a clear preference for a normal vaginal birth with minimal interventions [28, 33–37, 39–44, 46]. Church [28] identified that a normal vaginal birth was perceived as providing a sense of control over the birth experience. A normal vaginal birth was considered by midwives as a “state of being in control”; a state “in which the mother assumes some physiological power over the birth” ([28] p234). For some midwives in the literature, their professional experience of witnessing “awful normal deliveries” ([28] p233) led them to choose an elective caesarean [28, 38]. Again, Church’s study identified that this was related to control over preventing potential negative outcomes or complications associated with a normal vaginal birth [28].

#### Choosing a carer

The review identified that insider knowledge also influenced their choice of carer. There were two factors involved in the midwives’ choice of carers: sharing a similar midwifery philosophy with their carer [35, 40, 41] and needing to trust their carer [33–35, 37–39, 41, 44, 46].

##### Sharing similar philosophy and beliefs

Having similar birthing beliefs and philosophy to their carer was important to midwives’ when choosing their caregivers [33, 40, 41]. As one midwife described “I had found the person with whom I almost perfectly shared a philosophy and practice style…”([41] p26). Another midwife looked for midwife carers who would be comfortable in supporting her wish to have a vaginal birth after caesarean (VBAC) at home [40]. For another midwife her choice of obstetrician was based on choosing one that would not argue with her idea of a homebirth [35].

##### The need to trust their carer

Trust was an important element of support, and achieving a trusting relationship between themselves and their carer appeared to be crucial [33, 37, 41, 46]. In four of the articles reviewed, midwives talked about intentionally seeking out carers whom they felt they could develop trust with [33, 37, 41, 46]. For some midwives this meant having midwife friends and or colleagues with whom they already had a trusting relationship, to be present with and support them during labour, [34, 35, 37–39, 44]. Good support was important to achieving the type of birth the midwives wanted [33, 37, 38, 44, 46]. “I enlisted two close friends who I knew would be effective birth supports and looked forward to a vaginal birth” ([34] p48).

#### Choosing the model of care

In six of the reviewed articles, midwives identified a preference to be cared for under midwifery continuity of care models [34, 35, 37–39, 44]. Midwifery-led continuity models are those where a known midwife or small team of known midwives provide care and support throughout the antenatal, labour and birth, and postnatal periods [47]. Midwives in this review sought out care options as soon as possible to ensure they got care from a midwife they knew and trusted [33].

#### Choosing to birth at home

In twelve of the reviewed articles, midwives discussed wanting to have a home birth [28, 33–35, 37, 39–44, 46]. For several midwives this choice was because they believed giving birth at home provided them with a sense of control and autonomy over their birth experiences [33–35, 40, 42, 44, 46].

Having insider knowledge as a midwife gave midwives knowledge about homebirths including knowing that at home, they would be free to labour how they wanted, with privacy and without unnecessary hospital interventions [28, 33, 43, 44]. For some midwives their professional experiences of childbirth in hospitals, as well as their involvement in obstetric emergencies, led to a perception of a higher chance of these emergencies occurring in hospital [35] and thus there was a perception that they would experience a greater loss of control birthing in the hospital setting [33, 35, 44].

### Theme 2: Navigating the childbearing journey

The theme ‘Navigating the childbearing journey’ included three subthemes: ‘taking advantage of insider knowledge to achieve agency’; ‘difficulty achieving agency’; and ‘managing the midwife brain – the internal dialogue’.

#### Taking advantage of insider knowledge to achieve agency

Insider knowledge not only influenced midwives’ decision-making; it was also used to navigate the maternity care system. Midwives described drawing on their professional knowledge and professional status at times, to challenge the system and those working within it, to achieve their birth goals [28, 34, 35, 38]. As such, insider knowledge was seen by some as providing midwives with an advantage in achieving what they wanted and gaining control and support for their decisions during the birth [35, 37, 38]. In the postnatal period, insider knowledge of breastfeeding, and professional experience supporting women, was seen as an advantage in navigating their own breastfeeding journey. In McMulkin and Malone’s [31] descriptive cross sectional study of 210 midwives’ personal experience of breastfeeding, 66% felt that the theory they learned about breastfeeding was applicable to practice, and of these 76% felt that this theory was applicable to their personal experience of breastfeeding. For the midwives in this study [31] their experience helping mothers learn to breastfeed played a positive role in their own ability to breastfeed.

#### Difficulty achieving agency

While some midwives described how their professional standing as a midwife was used to successfully negotiate care, not all midwives were treated as knowledgeable decision makers, with some experiencing difficulty in achieving agency [28, 31, 34]. Despite having professional midwifery knowledge, some midwives described how their ability to be actively involved in the decision-making surrounding their care was difficult [28, 34]. For these midwives, maternity care providers assumed greater control over their births, and this was particularly evident in the midwives who birthed in hospital [28, 31].

#### Managing the midwife brain – the internal dialogue

While midwifery knowledge gave some midwives agency, insider knowledge and experience was a double-edged sword. Midwives experienced a constant internal dialogue during their pregnancy between their midwife brain and the childbearing woman’s brain [34, 35, 39, 41, 44]. This internal dialogue had both positive and negative consequences. For some it provided reassurance but for others it created anxiety.

##### The midwife brain creates fear and anxiety because of what they know

For some midwives the internal dialogue appeared to be driven by their knowledge of potential complications and negative outcomes. This meant they were on the lookout for things that could go wrong [28, 35, 39, 44] and this contributed to fear, stress and anxiety [28, 35, 37, 38, 41]. For example, one midwife described experiencing feelings of fear during labour, that her pelvis was not large enough to let the baby through [37], and another expressed a fear of haemorrhage (because of fibroids) if she gave birth at home [28]. For other midwives’ their experience of caring for mothers who had had inductions caused anxiety around the potential need for intervention for their own labours [28, 38, 46].

##### The midwife brain creates tension between a pregnant woman’s brain and the midwife’s brain

For some midwives, the internal dialogue caused by having insider knowledge caused tension between a pregnant woman’s brain and the midwife’s brain. Constable [35] described this as an internal argument within her own brain:

“At 17 weeks I had a small post-coital bleed and this was the first time I really discovered the internal argument between the midwife part of my brain and the paranoid pregnant woman side” ([35] p15).

In Constable’s description, the midwife brain would present logical reasons for a haemorrhage, while the pregnant woman’s voice would say "but what if…"([35] p15). Jennings [39] described herself as the “anxious pregnant midwife” ([39] p19) relating a discussion with her husband about whether to go to hospital to have a cardiotocograph, despite recognising that as a midwife she would not recommend this to a woman in the same situation [39]. This internal argument could cause conflict for midwives, who were caught between their midwifery knowledge of things that could happen and their instincts to just labour and birth [28, 35, 39, 44].

‘As my birth approached, I was caught between my knowledge of all the things that can happen, my belief that attended birth is safer and my instinct to crawl into a hole and have my baby without anyone around” ([41] p26).

This conflict was seen as negative for some to a point where one midwife wished she didn’t have insider knowledge.

“…when I had that bleed …. that was the one time when I thought ‘oh gosh, I wish I didn’t know what I know, because I think I could, you know, imagine all the problems that were going on with that bleed’….” ([28] p233).

Comparing her pregnancies, Constable [35] described her pregnancy after becoming a midwife, as being more anxiety driven and needing the psychological reassurance of listening to the heartbeat.

##### The midwife brain creates reassurance

Although insider knowledge could cause a negative internal dialogue between the pregnant woman and pregnant midwife, insider knowledge was also seen by some midwives as positive. For these midwives their professional knowledge was reassuring because they knew the most likely cause of small problems that arose [25, 28, 29, 35, 44]. For others, their knowledge helped to prepare them for possible outcomes, providing insight to accept situations when obstetric risks outweighed the possibility of a safe normal birth [25, 28]. There were also elements of their care, such as vaginal examinations, that they were able to perform on themselves because of their midwifery skills. For example, several midwives examined themselves vaginally to determine their own progress in labour [39, 40, 42, 44].

Midwives also used their professional knowledge and experience to reassure themselves in the postnatal period. In a study of midwives’ knowledge and attitudes towards breastfeeding, despite a large number of midwives reported experiencing problems with breastfeeding, these were not necessarily perceived as unresolvable, but rather viewed as part of the process that with persistence could be overcome [29].

### Theme 3: impact of care on the birth experience

The theme ‘Impact of care on the birth experience’ included two subthemes. These were: ‘positive relationships lead to positive experiences’ and ‘disrespectful care may lead to birth trauma’. In the reviewed articles, midwives described various experiences of care.

#### Positive relationships lead to positive birth experiences

Midwives reported that when they achieved trusting and respectful relationships with their maternity carers, they viewed these in a positive way. They described their maternity carers as being fully focused on them [33], made to feel special [46] and “cocooned” in love ([40] p60).

Having a positive relationship with their maternity caregiver appeared to be equally important regardless of where the midwife gave birth. The majority of midwives who had homebirths reported positive relationships with their caregivers, were satisfied with the care they received, and reported an overall positive birth experience [33, 35, 37–40, 42–44, 46]. Midwives who gave birth in hospital and who reported a positive relationship with their maternity caregivers, also reported an overall positive birth experience [36, 38, 45].

Those who described positive relationships with their carer described trust as being crucial to their relationship. In the reviewed literature, trust was expressed in two ways: the carers’ trust in the childbearing midwives’ ability to give birth, and the childbearing midwives’ trust in their carers commitment to honour and respect their wishes. Midwives expressed the need to have a mutual trust so that they could feel relaxed and comfortable [33, 37, 41, 46]. They felt that caregivers who trusted in their ability as a woman to birth their baby, was important to them to be able to trust in their own ability to give birth [33, 37, 46].

“When I lost faith during transition I needed, as an anchor, Sarah's quietly expressed belief in my body and the birth process: she held my belief for me when I could not” ([37] p25).

Trust was important for midwives during childbirth [33, 37, 38, 41, 46]. Having trust in their carers meant the midwives were reassured that their caregivers would not intervene inappropriately, therefore providing them with a sense of control [37]. Consent appeared integral to trust, with midwives stating that nothing was done to them during labour without consent [38] and everything was consented to, including vaginal examinations [33]. Achieving a trusting relationship with their carers also meant they were able to ‘let go’ of their midwife brain during the birthing process and let their body do what it needed to do to give birth [33, 37–39, 42, 44]. One midwife described this letting go as being able to “slip out of the neo-cortical activity of the intellect that Odent writes about and into another consciousness” ([37] p24). Letting go was also described as “that hormonal fog of active labour” ([39] p20) and “disengaging the mind” ([44] p14) which allowed the body to take over and push their baby out.

Midwives who achieved trusting and respectful relationships with their caregivers described giving birth as being an empowering [43, 44] and powerful experience [37, 41, 42, 46]. Their birth gave them confidence [33, 44] and a great sense of achievement [37, 45] at being stretched to their limit and finding out they are more than what they thought, providing a sense of completeness about the birth experience [33]. Others described the experience as being awesome [37], fulfilling [43], exhilarating [39], and an “explosion of fireworks” (45 p62). One midwife described her experience as a reminder that the body is truly awesome and intrinsically designed to birth [44].

#### Disrespectful care may lead to birth trauma

Conversely, not all midwives described a positive experience with their care provider. Some described feeling dismissed by their care providers, vulnerable, in need of more support, and or receiving indifferent care. Feeling dismissed by caregivers was often within the context of the lack of a mutual trusting relationship. While this was mostly reported within the hospital setting, Burlow [34] described her request for a homebirth as being faced with opposition from the community midwives who were based in the hospital, ultimately leaving her feeling “trapped” and “powerless” ([34] p18).

This review identified that for most of the midwives who birthed in the hospital setting, fears and anxieties, heightened by their increased professional knowledge, were often unaddressed by caregivers, even at times having their valid concerns disregarded and dismissed [25, 28, 46]. These midwives were left feeling alone [28, 31, 41], unsupported in their worries [28, 31, 46] and with the feeling that their professional knowledge was unacknowledged [28]. Midwives were also anxious about possible negative outcomes for their babies. This was exemplified in one midwife’s story of being dismissed by her caregivers when she presented with premature rupture of membranes, only to find it confirmed by a scan weeks later. Her fear and anxiety during this time was not addressed by maternity caregivers, which left her feeling “cross,” ([28] p233) dismissed, and anxious about the possible negative outcomes to her baby [28].

When it came to breastfeeding, midwives who gave birth in hospital mostly reported being left to their own devices with little support from their care providers [31, 46]. Studies found that being unsupported in breastfeeding was due to their professional status as midwives [31, 46]. McMulkin and Malone’s [31] study on breastfeeding support reported that the midwives were “… respected by the staff as professionals who didn’t require any assistance or support” ([31] p12) with breastfeeding and the care of their new baby [31]. This approach to breastfeeding support could also be considered dismissive, because some midwives in their study described feeling ‘helpless’ ([31] p12) when confronted with their own baby, and stated that they would have appreciated more support than they received in the early stages of breastfeeding their baby [31].

While some midwives reported a lack of adequate support, another midwife described receiving an indifferent approach to care [46]. This midwife described feeling like she was on a conveyor belt and that “we were simply 'going through the motions” ([46] p25). She met the midwife only on arrival to hospital and never felt completely relaxed and safe during her transitional stage. She described not feeling encouraged or supported:

“I never felt completely relaxed or safe, and during the transitional stage, rather than feeling encouraged and supported, supported in feeling the overwhelming emotions which overtook me, I felt that I must do as I was told and not ‘lose control’” ([46] p25).

The effect of this was that she had “no memory of euphoria, no warm words of congratulation between us and our midwife, simply paperwork, a goodbye and a shift change!” ([46] p25).

Ultimately, midwives who received respectful care and developed a trusting relationship with their care provider often reported overall positive birth experiences [33, 36–40, 42–46], whereas those who were not treated with respect felt their birthing experiences were negative or traumatic [25, 28, 31, 32, 34, 46].

##### The impact of birth trauma

In six of the reviewed articles, midwives reported feeling traumatised by their experience of childbirth [25, 30, 32, 34, 40, 41]. These midwives described having flashbacks, nightmares and panic attacks for years [40]. In the study by Toohill, et al. [32] 97 (41.6%) participants indicated they experienced trauma during their labour and birth. Having interventions was the main cause of trauma, and receiving maternity care which was described as ‘assaulted’, ‘aggressive’, ‘demeaning’, ‘intimidation’ and ‘bullying’ ([32] p67) were also key reasons given for birth trauma. In another study, more than one-fifth of midwives (22.%) reported a traumatic experience when giving birth themselves [30] and this (OR = 1.76, 95% CI [1.09, 2.83]) was associated with an approximately twofold increase in risk for probable PTSD. Having a traumatic birth experience was also associated with Postnatal Depression (PND) [25]. Of the 22 participants in the study by Redwood [25], six were diagnosed with PND and three exhibited symptoms of PND during the interviews.

Depression was also reported by midwives in three of the reviewed articles [25, 32, 45]. Being separated from their baby was seen as contributing to developing depression [32] and this was closely tied to difficulties with bonding with their baby [25, 32, 45].

### Theme 4: From midwife to mother

The theme ‘From midwife to mother’ included two sub-themes: ‘unprepared for childbirth and motherhood’ and ‘transitioning to motherhood’.

#### Unprepared for childbirth and motherhood

In several of the studies midwives expressed that their professional knowledge and experience did little to prepare them for childbirth and motherhood despite their expectations that it would [25, 41, 45]. For one midwife who’s birth had not gone according to plan, she declared “never in a thousand years would I have anticipated this happening to me” ([34] p18).

Despite caring for labouring women on a regular basis, many midwives described the shock of labour pain [25, 33, 37, 41], and used words such as “horrendous” ([36] p4) “bone-crushing”, “overwhelming”, “unbearable”, “exquisite pain” ([41] p26) that was “so terrible and so frightening” ([37] p23).

For some midwives, the pain was “horrendous” and “shocked” beyond all expectations ([33] p5) with some left wondering why no one had told them about how painful it would be [25, 37]. Lee-Ribas [41] had “no idea” of the “bone crushing, unbearable, overwhelming” and “exquisite pain” of “back labour” ([41] p26) but her realisation that she hated giving birth and having the “veil of illusion about birth” ([41] p27) being torn away was as painful as the physical labour. Her experience of birth, left her questioning how she could have supported so many women without realising what they were going through.

“How could I, how could I have stood beside those women, so many of them, calmly breathing, murmuring ‘you can do it. You are doing it,’” “Letting them trust me. I knew nothing. I did not deserve their trust” ([41] p27).

Hinsliff [38], although able to understand the reasons for the procedures she had during labour, described labour as a “terrifying experience” ([38] p48) from the second stage onwards.

In addition to not being prepared for the reality of labour, midwives also experienced a conflict between their professional knowledge and their personal experience of breastfeeding. Tennant [45] described having a very difficult and unexpected breastfeeding experience and felt that her midwifery training did not prepare her for motherhood, despite her expectations to the contrary.

“My experience as a midwife did very little to prepare me for motherhood. Did I expect it to? Well, yes, on reflection I thought it would. The first day at home with my son James made me realise how mistaken I had been!” ([45] p438).

#### Transitioning to motherhood

The quality of their birth experience and transition to motherhood appeared to be dependent on their perceived level of control during childbirth. Control was largely contingent on the level of effective communication and trust experienced between themselves and their maternity care providers [25, 33, 37, 46]. In Redwood’s [25] study, even when birth did not go to plan, participants who felt they were in control of their birth experience, and perceived that communication with their maternity care provider was good, had a better transition to motherhood than those who didn’t [25].

## Discussion

From the six research and 14 anecdotal papers reviewed, it appears childbearing midwives are a unique subgroup of women who have specialised knowledge, and a set of professional experiences that play a significant role in their childbearing journey. This review identified the potential conflict experienced by childbearing midwives, between their position as knowledgeable experts in maternity care, and as mothers. In some instances, they were able to use their insider knowledge about models of care, and benefits and risks to make decisions and navigate the maternity care system. This insider knowledge, however, could also generate heightened fear and anxiety through pregnancy.

### Gaining control through knowledge and experience

Similar to other studies exploring women’s childbearing experiences, having control during their pregnancy and birth appeared crucial to the midwives and was connected to having an overall positive childbearing experience and optimal transition into motherhood. To remain in control, and to remain involved in the decision-making process about their care, midwives drew on their experience and knowledge to negotiate with their care provider. Midwives who felt they remained in control of their birth experience often had continuity of care from known care providers where positive and trusting relationships developed. Regardless of model of care, midwives who reported a positive relationship with their maternity caregivers also reported an overall positive birth experience [36, 38, 45].

The type of communication and care from maternity caregivers has been shown to either enable or prevent women from being actively involved in decisions about their birth [48, 49]. When women have been involved in the decision-making process, they generally report feeling positive and more in control of their birth experiences, even when their births do not go to plan [50, 51]. Thus, having a positive relationship with the maternity care provider, and subsequently feeling in control of the birth, are important to women perceiving their birth experience as positive [48].

### Feeling dismissed and unsupported

Some midwives who gave birth in hospitals were not always treated as knowledgeable decision makers, and therefore had great difficulty feeling in control of their pregnancy and birth care. This was exemplified when midwives’ opinions and valid concerns about their own personal situations were dismissed [28]. Some midwives also felt unsupported when they were expected to know what to do when it came to breastfeeding their new baby and would have appreciated more help. A study of American postpartum nurses also found they received less breastfeeding support than they needed, attributing this to the fact that they were postpartum nurses and expected to know what to do [52].

Women who are not midwives have also reported that they need more support from caregivers in the care of their newborn [53]. Burns et al.’s (2013) study on midwives’ discourses surrounding breastfeeding support for women, identified a discursive theme “breastfeeding – it’s not rocket science” [54] where breastfeeding was constructed as “natural” or “easy” ([54] p65), and something that all women could do if they were committed. This discourse resulted in women being left to their own devices, allowing midwives’ to attend to other aspects of postnatal care [54]. It is possible therefore, that when a woman is a midwife there might be an even greater assumption that they will be able to breastfeed and care for the baby due to their professional knowledge and experience.

### Midwives experience birth trauma too

This integrative review identified that midwives can experience birth trauma, particularly within the fragmented, non-continuity of care models within the hospital setting. Many women have also reported negative experiences of labour and birth care within the hospital setting describing the mistreatment they received from healthcare professionals as ‘barbaric,’ ‘intrusive,’ ‘horrific,’ and ‘degrading’ ([12] p2147).

Previous research has found an association between maternity care providers’ attitudes and approaches to care, and women’s experiences of childbirth [6–9, 12–16]. Women who did not feel well supported by their maternity care providers have reported their experiences as traumatic or dissatisfying [6]. Additionally, a lack of support from maternity care providers is related to postpartum anxiety [53] and poor support during labour has been identified as a risk factors for posttraumatic stress following childbirth [55–57]. It appears from this integrative review that midwives who give birth in a hospital setting, may also be vulnerable to the same kind of mistreatment that women in general report.

### Insider knowledge is a double-edged sword

Some midwives experienced heightened fear and anxiety about potential complications because of the insider knowledge they possessed, and their experience of caring for mothers. Although most midwives preferred a normal birth, for some the fear of potential complications with labour and birth led some to choose a caesarean birth [28, 38]. In this context, reliance on professional knowledge and prior experience appears to have influenced their thinking, as described by Edwards [58]. Edwards [58] claims that ‘obstetric thinking and practices have subjugated women’s concerns, power and strengths immeasurably’ with the consequences that such thinking is not necessarily even obvious to women [58]. The impact of obstetric thinking is similar to women where fear of complications that may occur during labour and birth and has been cited as a reason for choosing to have a caesarean birth [59].

### Caring for the midwife-mother

This review identified that midwives may experience a conflict between their desire to have their professional status respected, and their desire to ‘let go’ and fully be the birthing woman. The midwives who were able to ‘let go’ and give birth, while remaining in control, were those who had complete trust in their carer. However, for some maternity care providers there appeared to be a tension between seeing midwives as experts, and therefore leaving them alone, or as a challenge to the expertise of the maternity care provider. These findings are reflected in a recent study of healthcare professionals’ perceptions of caring for healthcare professional patients [60]. These participants felt that the best approach when caring for health care professional-patients was to provide responsive care which included acknowledging and respecting the patient’s identity as a healthcare professional, and responding to the patient’s wishes and how these were to be met [60].

When providing care for childbearing midwives, maternity care providers should consider the midwife’s unique set of knowledge and experience. Being aware of, and understanding, the potential conflict in roles that childbearing midwives may be experiencing means that maternity caregivers can better care for childbearing midwives by appreciating their heightened anxieties and respecting their knowledge. Svantesson et al. [60] recommend that healthcare professional-patients should be cared for just as any other patients would be, but, only if they are given ‘person-centred care’. It would then imply that providing true woman-centred care for women who are midwives means finding a balance between acknowledging the vulnerable woman in the professional midwife, and acknowledging the identity of the professional midwife in the woman [60].

### Limitations

This integrative review was only able to identify research from the United Kingdom and Australia, and the anecdotal articles of midwives’ personal birthing experiences from the United Kingdom and the Unites States of America. Therefore, the findings of this review are only representative of a very western perspective.

## Conclusion

The findings of this review, highlight the potential conflict for childbearing midwives between their position as knowledgeable experts in maternity care, and their experience as mothers. Whilst they are able to use their insider knowledge to their advantage, they can also experience heightened fear and anxiety through their pregnancy. It is important for maternity care providers to acknowledge and support them and provide balanced and tailored care that takes into account all that the pregnant midwife is: a professional midwife and a vulnerable child birthing woman. However, as the findings of this integrative review are based on limited research and anecdotal evidence, systematic and methodologically sound research is needed to gain a better understanding of midwives’ own experiences of birth, and the way their experience impacts on their practice. It is possible that by researching this topic, it can help inform practice by outlining how best to provide maternity care that supports this unique subgroup of professional women as they transition into motherhood. Further research into the personal childbirthing experiences of midwives is recommended.

## Data Availability

The datasets used and/or analysed during the current study is available from the corresponding author on reasonable request.
